# Supplementary material for: Quantitative confocal microscopy and calibration for measuring differences in cyclic-di-GMP signalling by bacteria on biomedical hydrogels
Source: R Soc Open Sci. 2021 Jan 6;8(1):201453. doi: 10.1098/rsos.201453 (PMC7890475; doi:10.1098/rsos.201453)
Supplement: Supporting Information for Quantitative confocal microscopy and calibration for measuring differences in cyclic-di-GMP signaling by bacteria on biomedical hydrogels [file rsos201453supp1.docx]

**Supporting Information for**

**Modulating the crosslinking of a biomedical hydrogel impacts cyclic-di-GMP signaling linked to biofilm formation**

A

B

PEGDA 10k Da

PEGDA 2k Da

**Supplemental Figure S1**

NMR spectra of (A) PEGDA 2k Da and (B) PEGDA 10k Da.


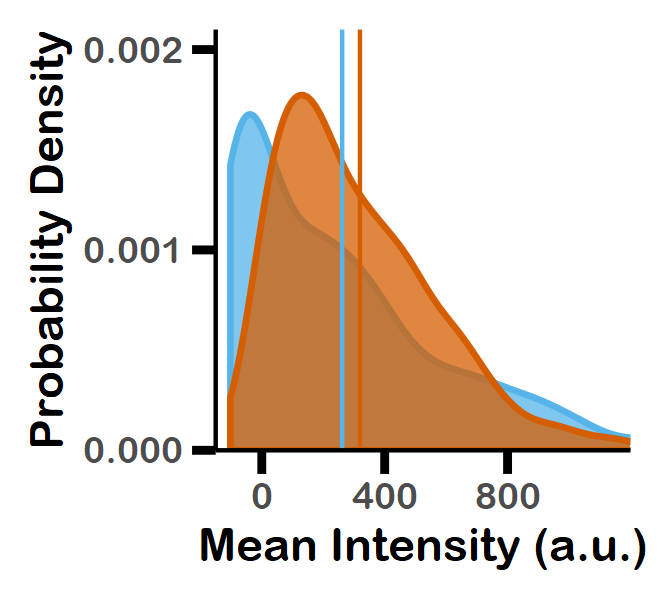

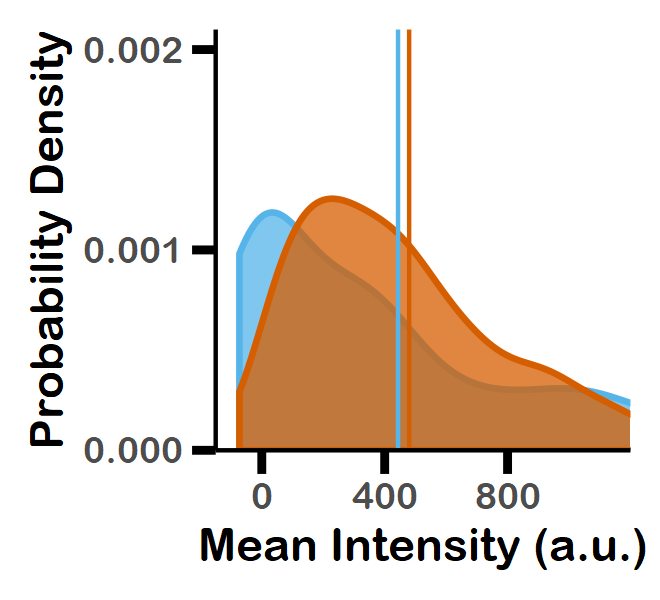

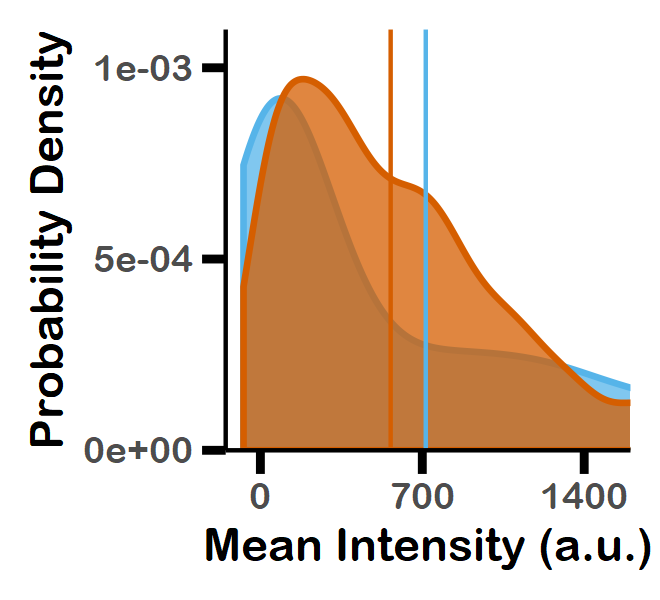

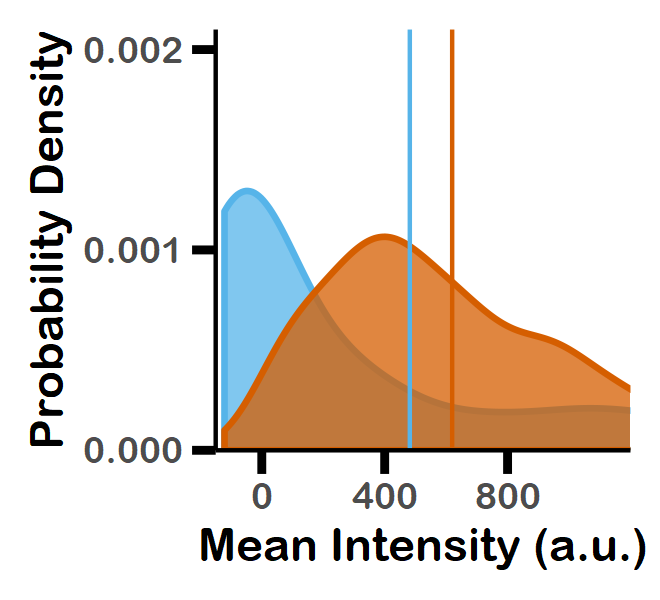

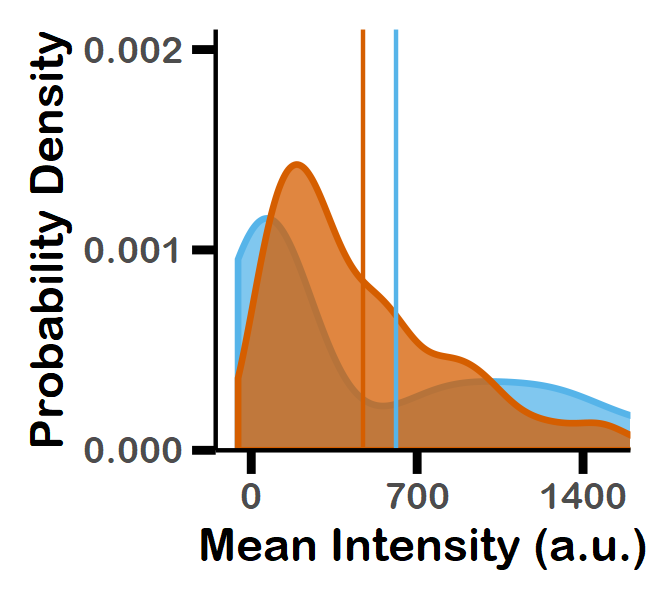

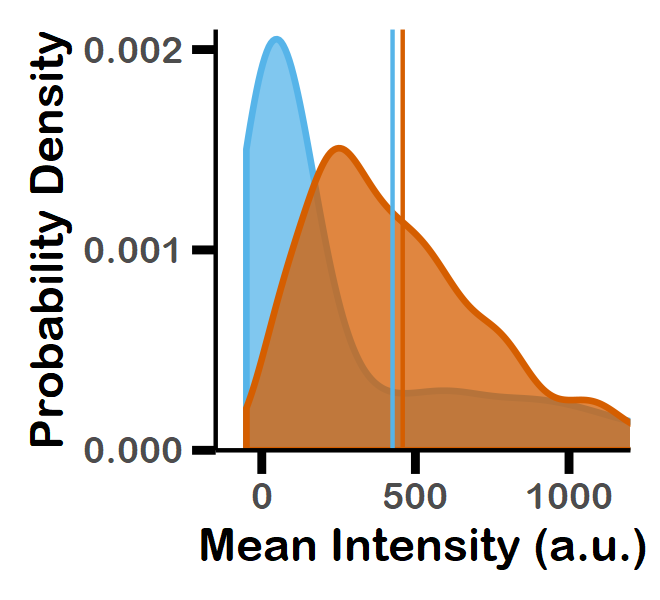

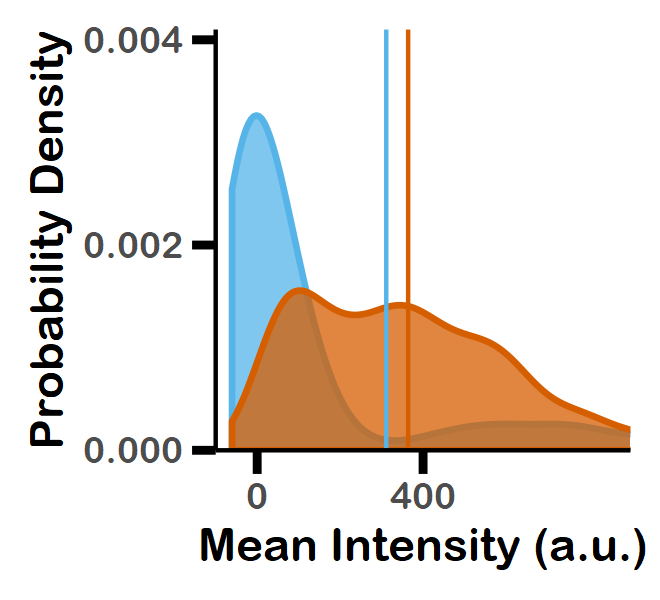


A

B

C

D

E

F

G


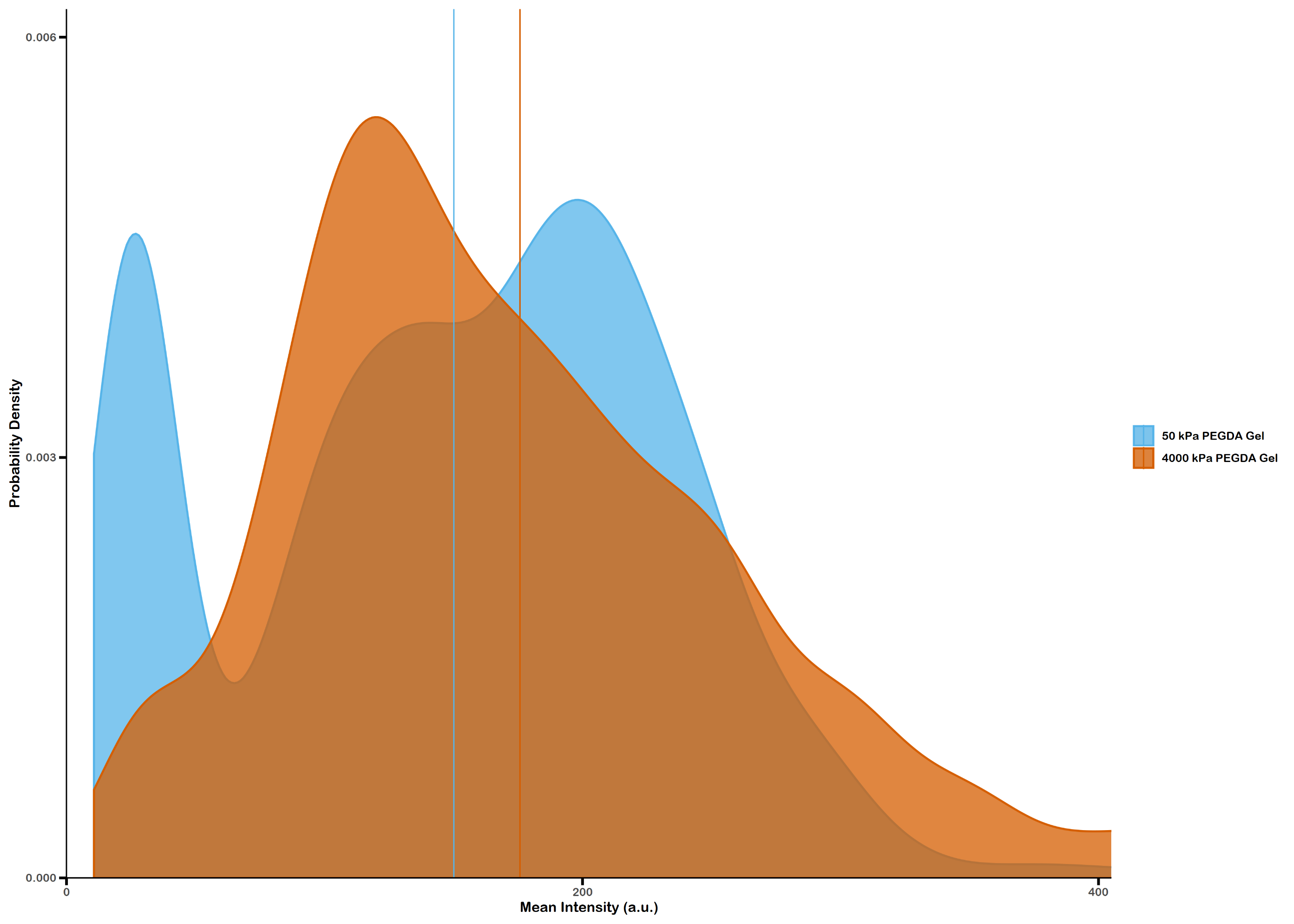


**Supplemental Figure S2**

The probability density functions of WT reporter pCdrA::GFP cells at (A) 60 minutes post attachment, (B) 90 minutes post attachment, (C) 120 minutes post attachment, (D) 150 minutes post attachment, (E) 180 minutes post attachment, (F) 210 minutes post attachment, (G) 240 minutes post attachment.


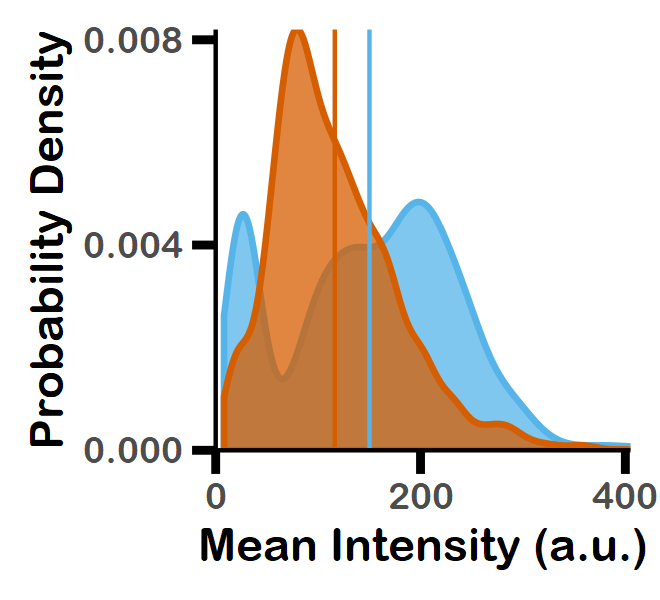

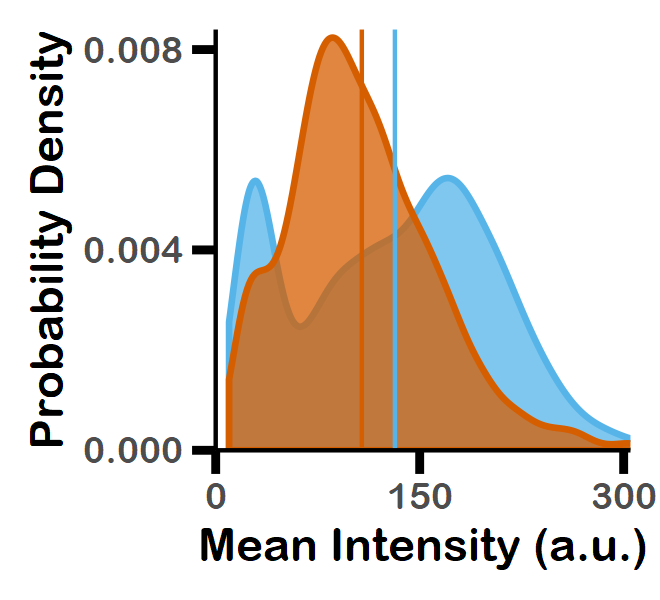

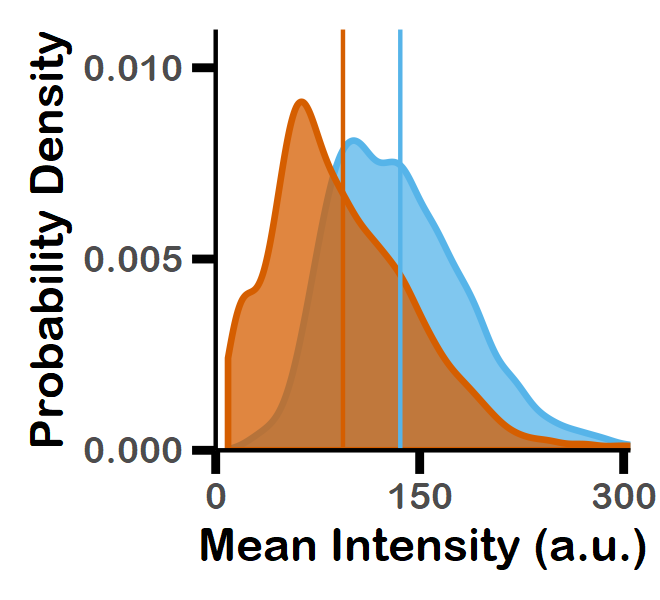

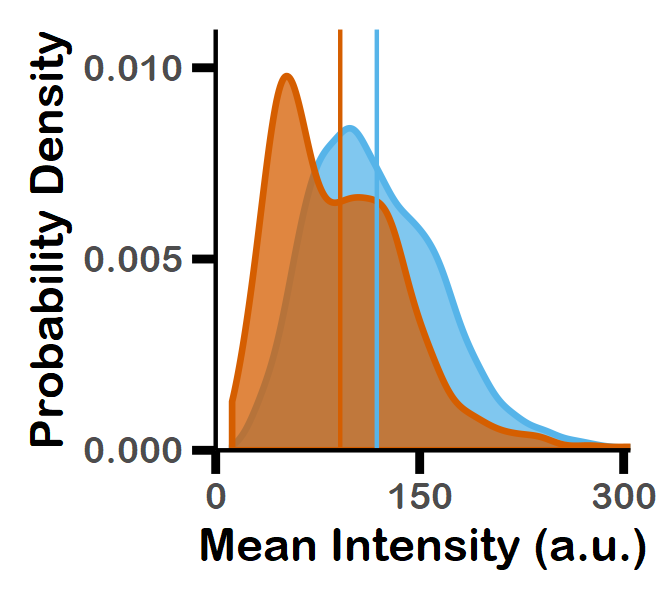

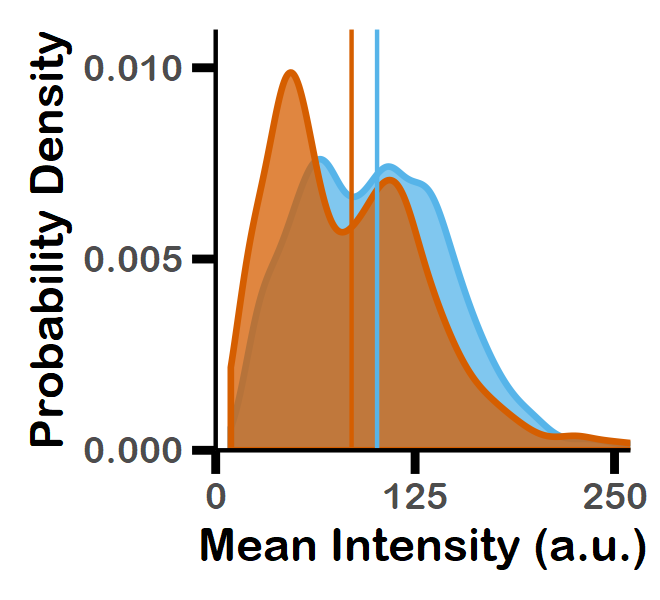

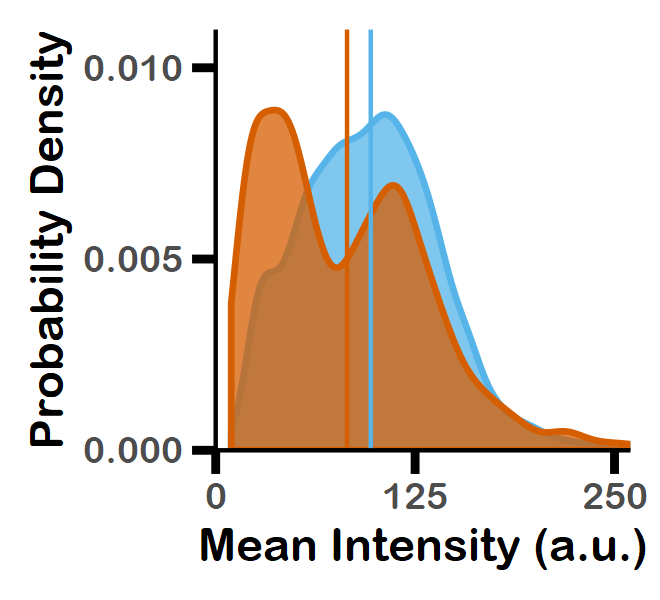

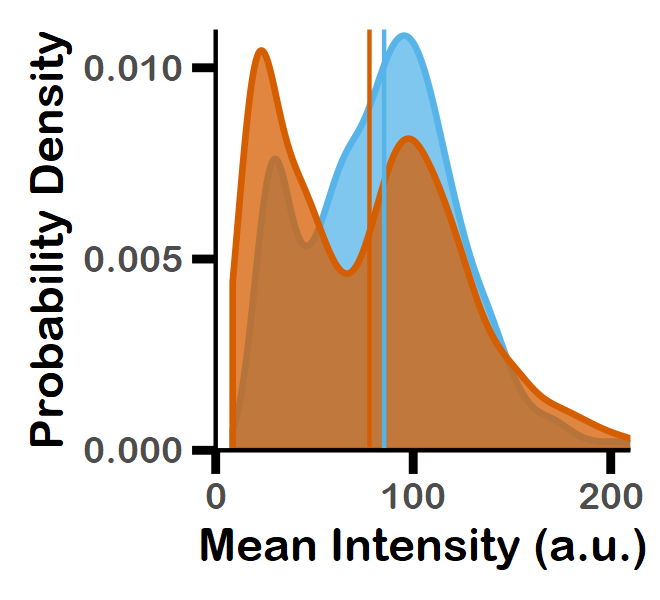


A

B

C

D

E

F

G


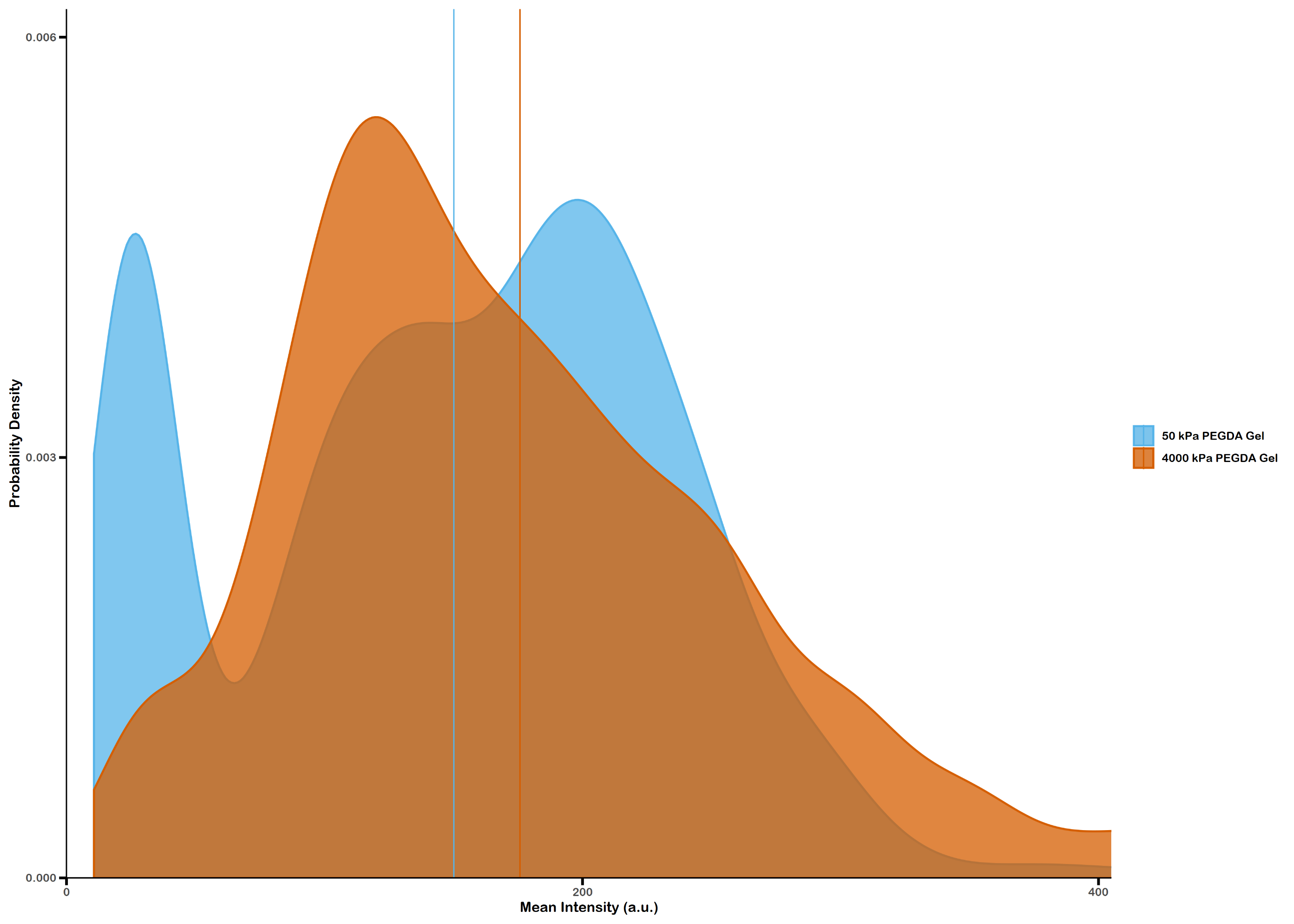


**Supplemental Figure S3**

The probability density functions of WT control pMH487 cells at (A) 60 minutes post attachment, (B) 90 minutes post attachment, (C) 120 minutes post attachment, (D) 150 minutes post attachment, (E) 180 minutes post attachment, (F) 210 minutes post attachment, and (G) 240 minutes post attachment.


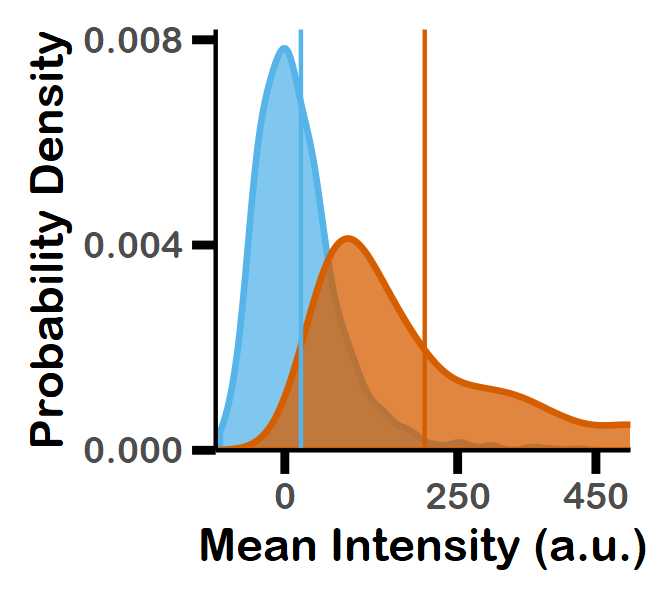

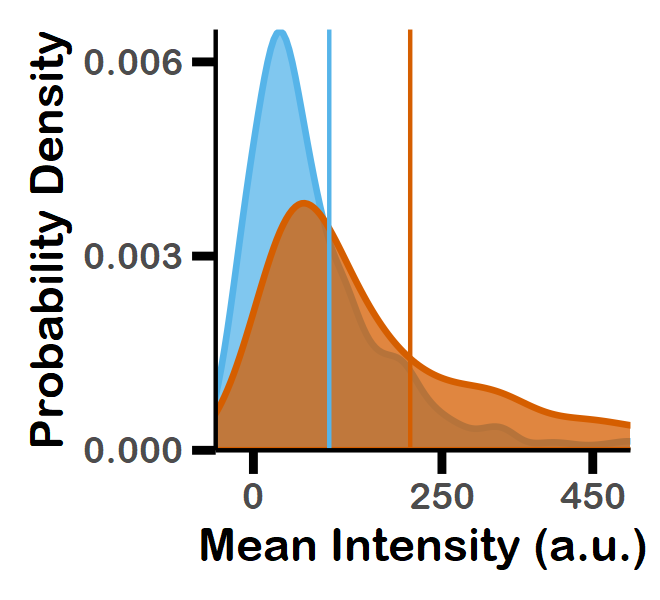

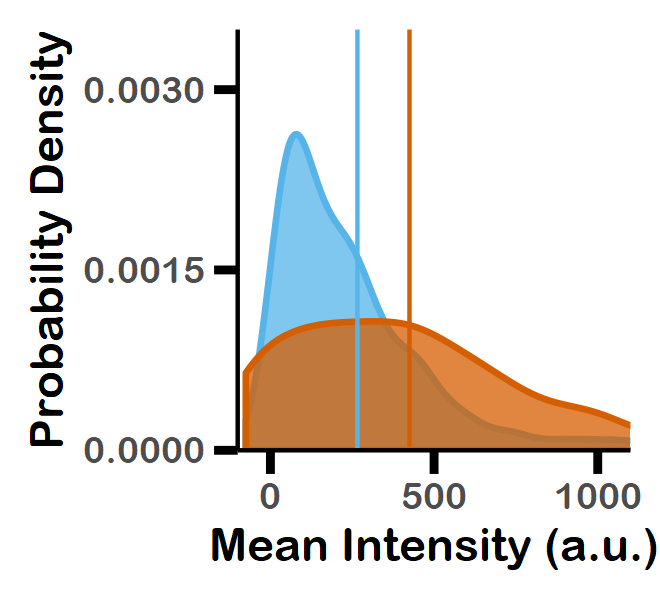

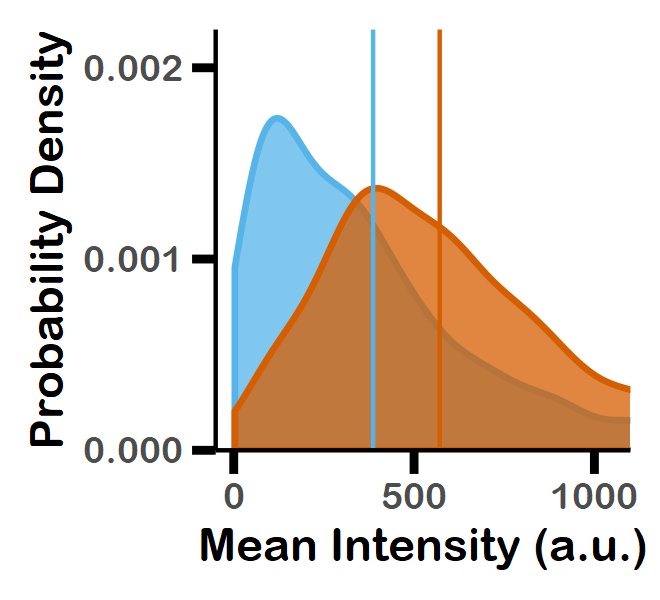

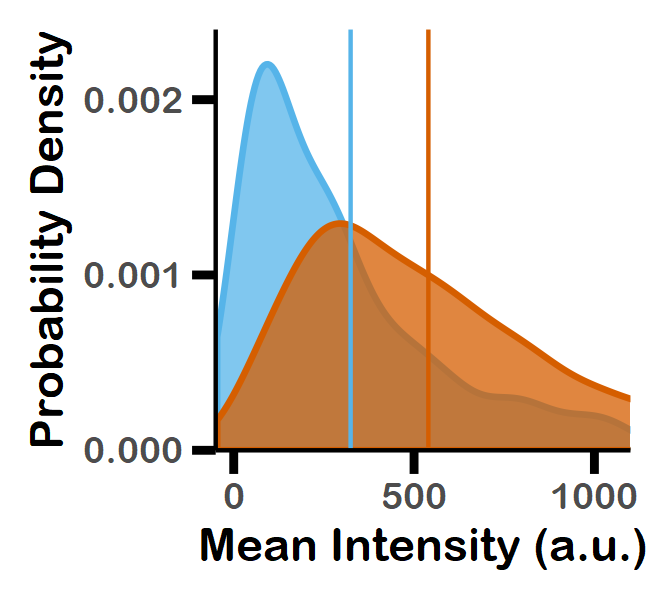

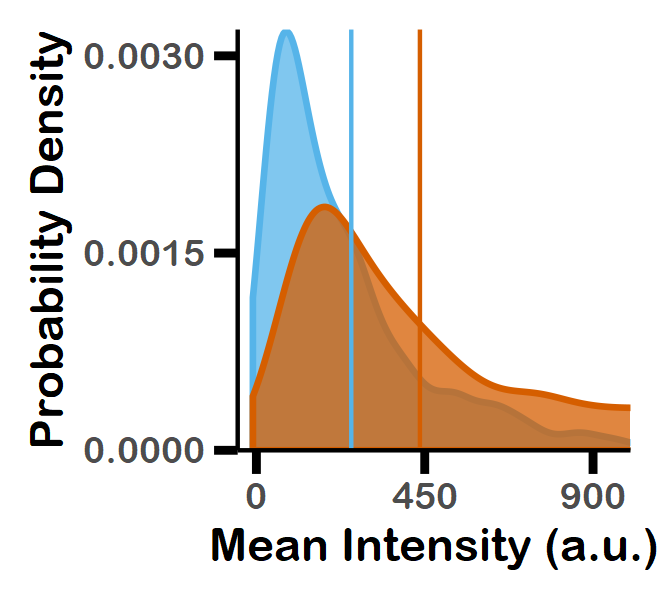

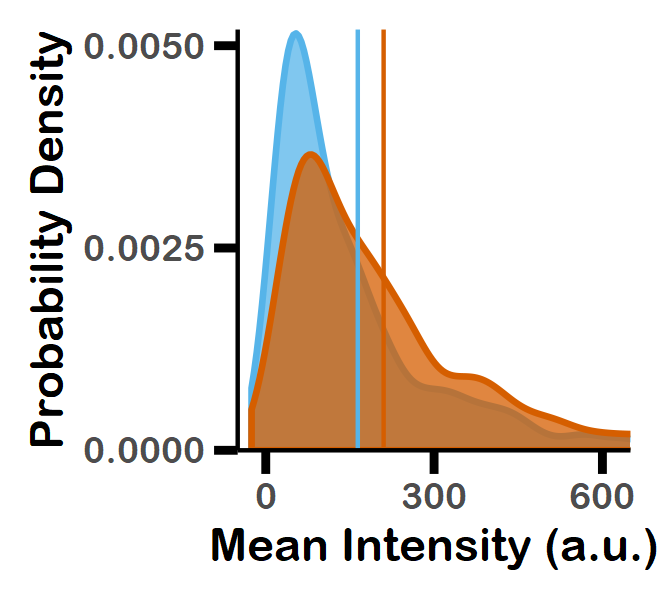


A

B

C

D

E

F

G


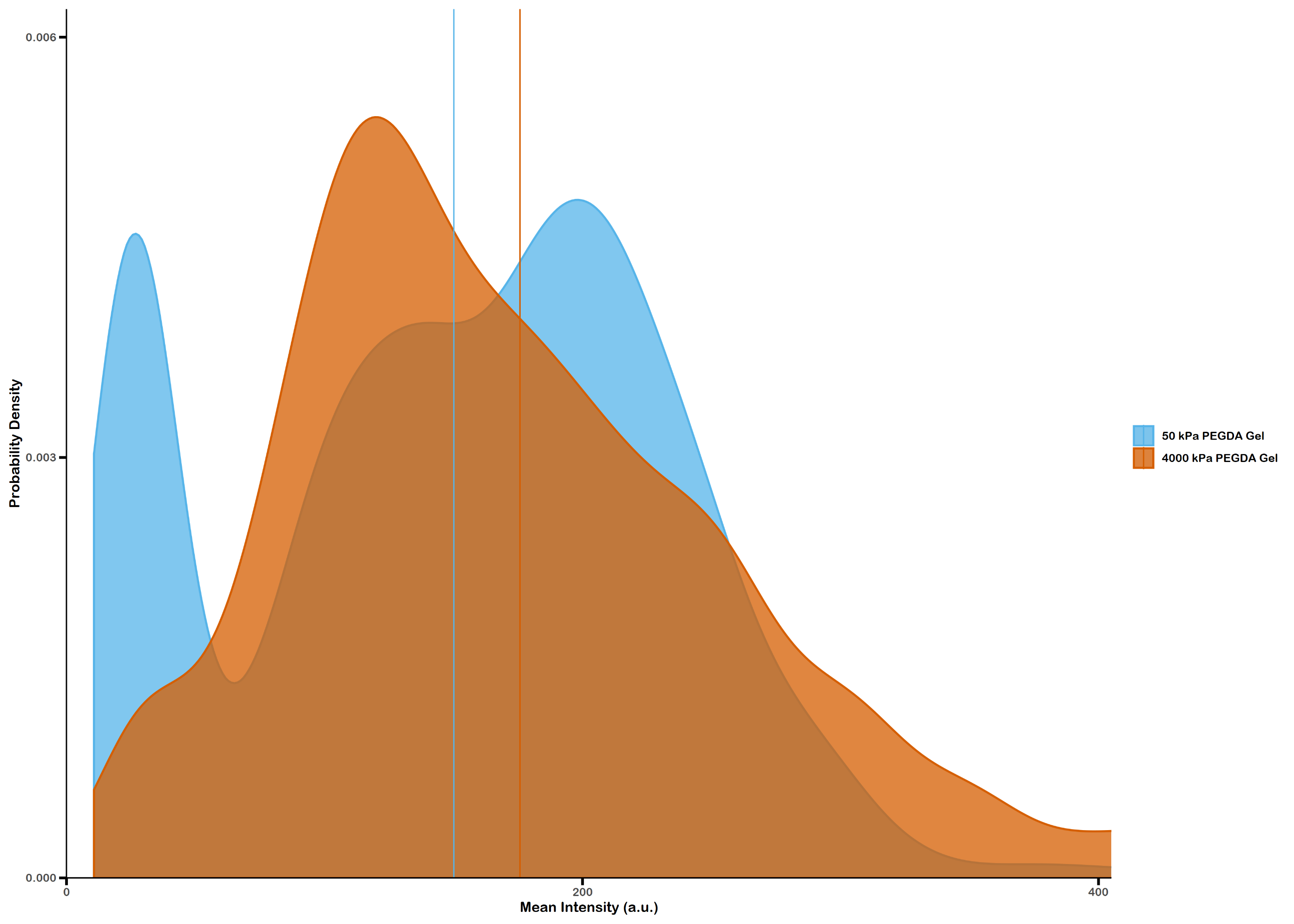


**Supplemental Figure S4**

The probability density functions of Δ*pily1* reporter pCdrA::GFP cells at (A) 60 minutes post attachment, (B) 90 minutes post attachment, (C) 120 minutes post attachment, (D) 150 minutes post attachment, (E) 180 minutes post attachment, (F) 210 minutes post attachment, (G) 240 minutes post attachment.


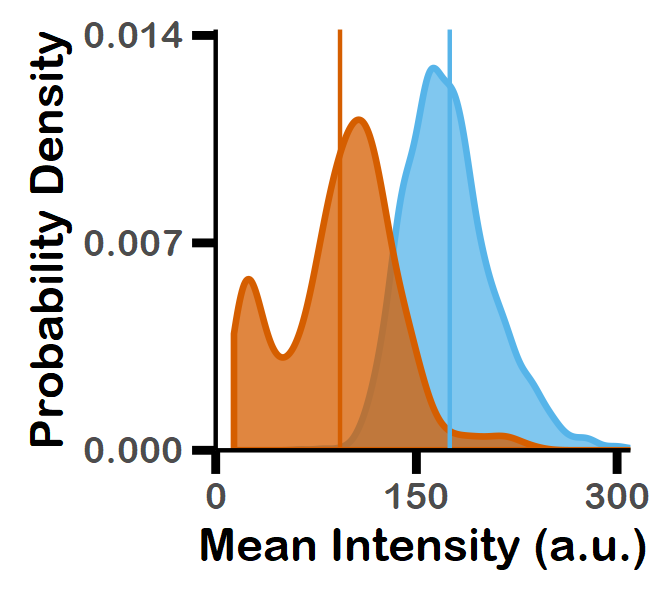

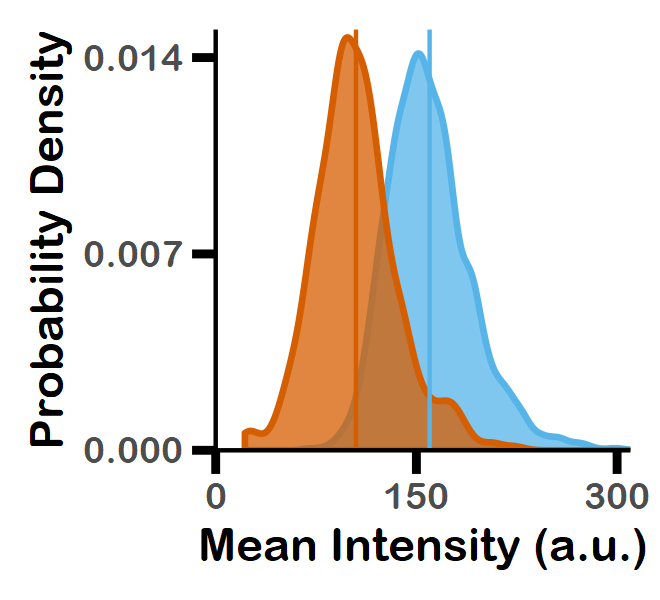

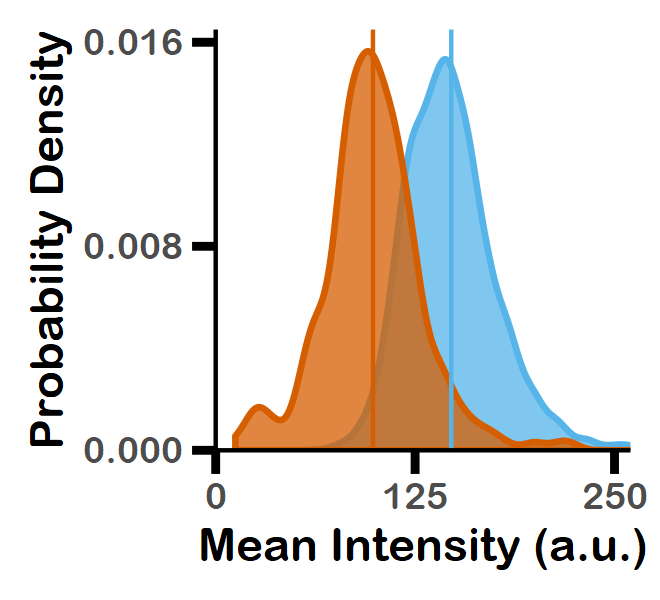

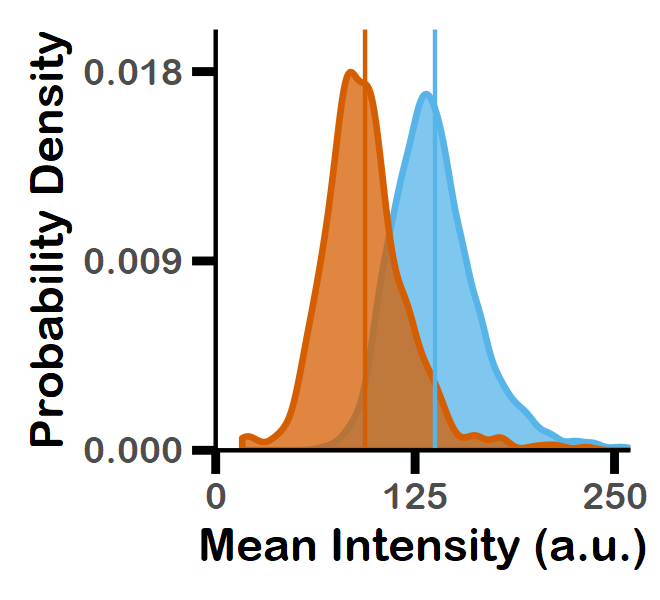

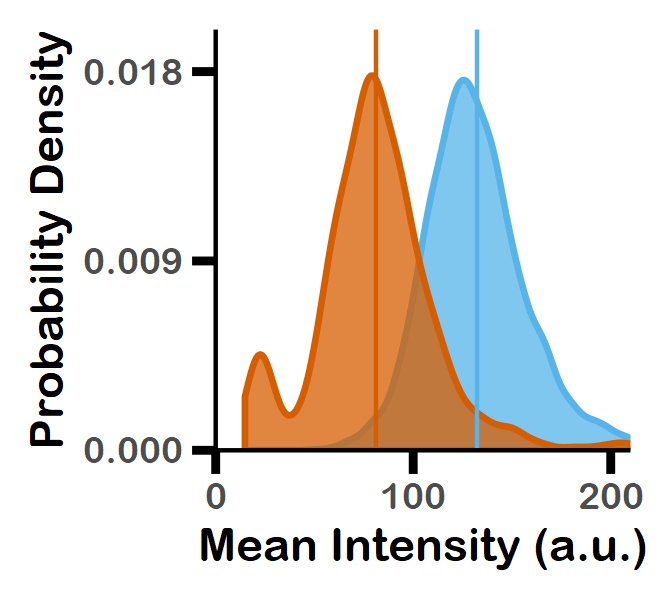

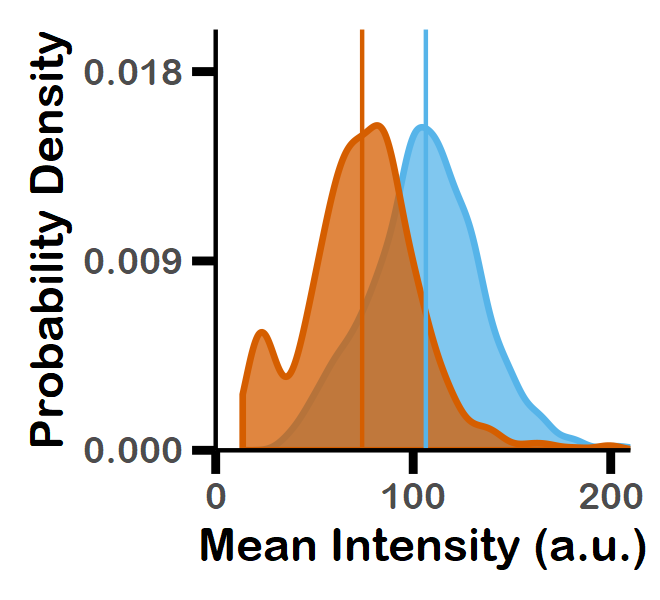

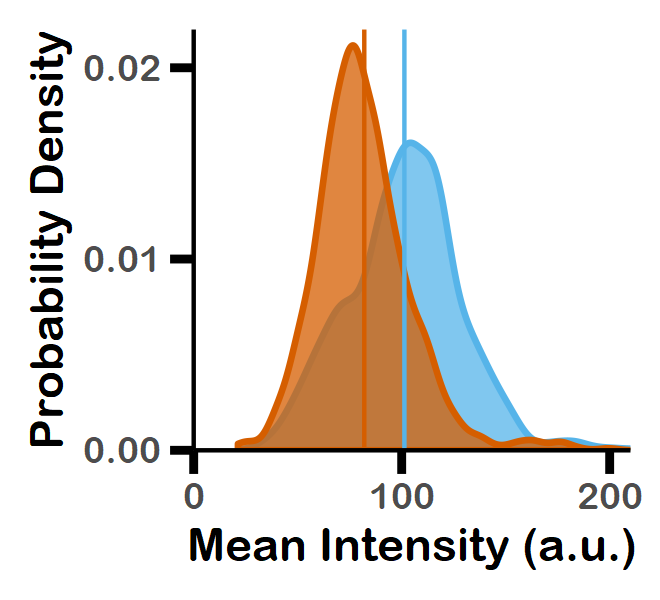


A

B

C

D

E

F

G


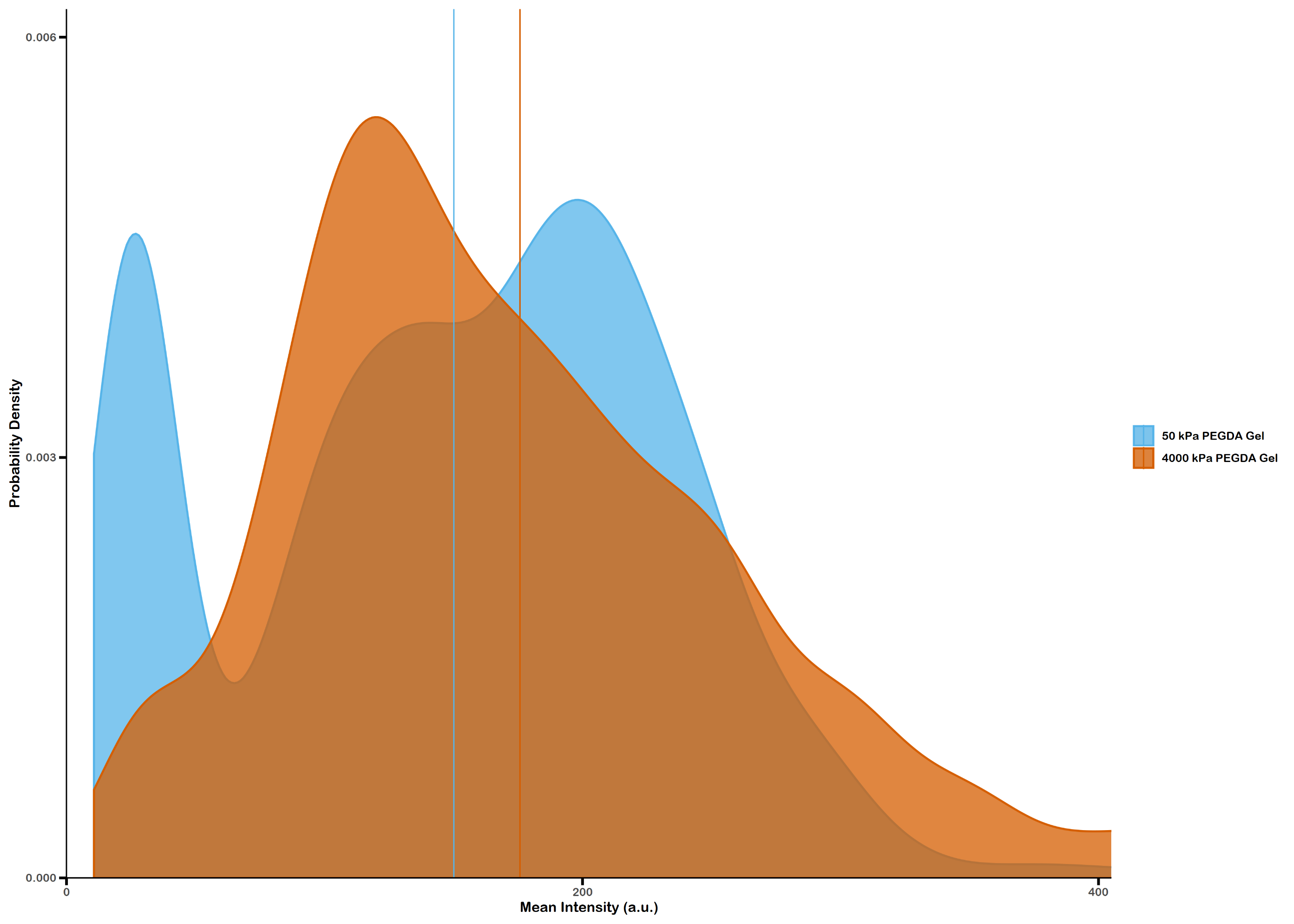


**Supplemental Figure S5**

The probability density functions of Δ*pily1* control pMH487cells at (A) 60 minutes post attachment, (B) 90 minutes post attachment, (C) 120 minutes post attachment, (D) 150 minutes post attachment, (E) 180 minutes post attachment, (F) 210 minutes post attachment, (G) 240 minutes post attachment.


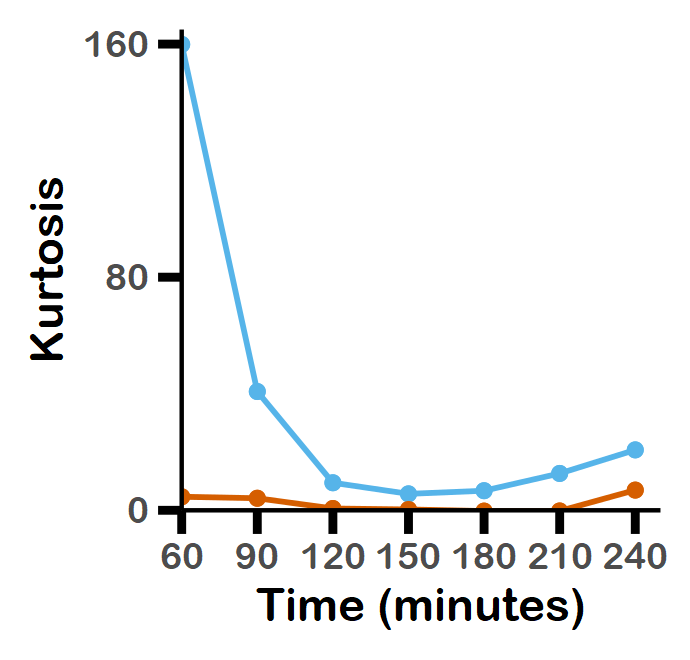

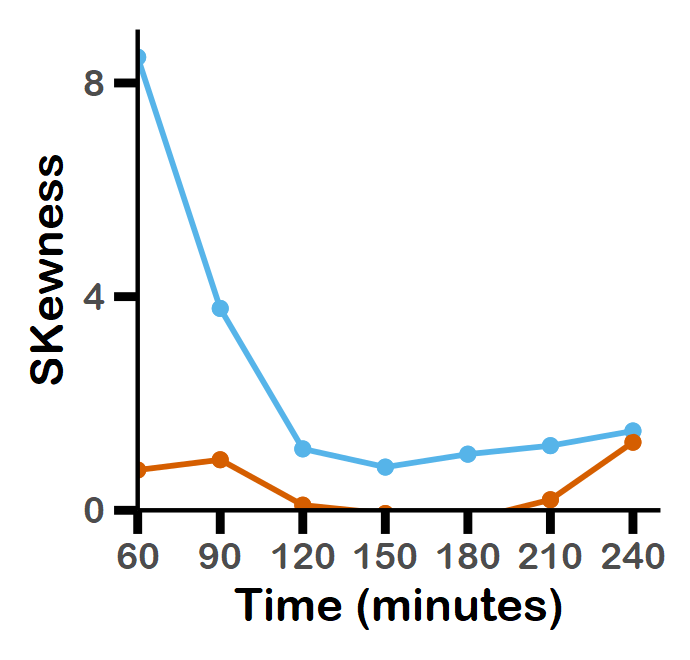


A

B


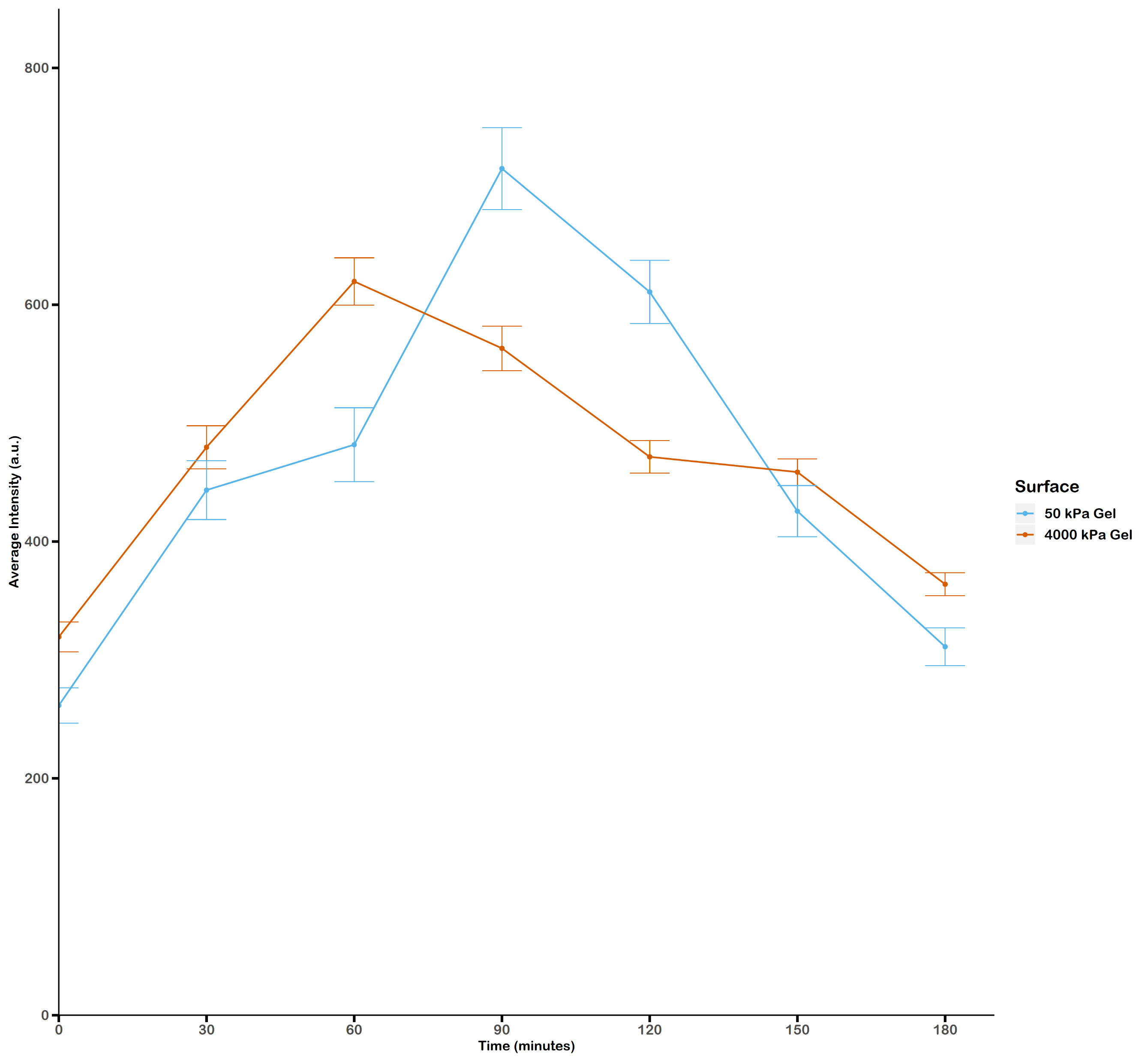


**Supplemental Figure S6**

The difference in skewness and kurtosis for Δ*pily1* and WT populations. (A) Δ*pily1* skewness minus WT skewness for every timepoint. The kurtosis of Δ*pily1* populations is higher than that of WT populations at all times except on the stiff gel at 180 minutes. (B) Δ*pily1* kurtosis minus WT kurtosis for every timepoint. The kurtosis of Δ*pily1* populations is higher than that of WT cells at all times except on the stiff gel at 180 minutes.
